# How the United States Is Meeting the Tuberculosis War Problem

**Published:** 1918-11

**Authors:** 


**How the United States is Meeting the Tuberculosis War Problem.** Colonel G. E. Bushnell, M. C. *The Military Surgeon*, Aug. 1918.

In little more than one year's time, according to the wish of the National Tuberculosis Association, the whole United States Army has been re-examined for tuberculosis.

Out of 1,406,498 men examined, 11,020 (0.783 per cent.) tuberculous subjects, or suspected to be tuberculous, were rejected.

The examination of the sputum is the only reliable means of distinction between both diseases. No soldier can henceforth be discharged for pulmonary tuberculosis, unless his sputum proves positive for tubercle bacilli.

Important provisions are being made by the Government for the care of the tuberculous soldier. Many sanatoria have been leased, many buildings erected and many hospitals organized to care for thousands of tuberculous patients. The William Wirt Winchester Memorial Tuberculosis Hospital at New Haven, Conn., is used as a training school for the staffs of tuberculosis hospitals and for tuberculosis examiners.

The same strict care as is enforced for casualties must be applied to the tuberculous. The best thing for the patient is to remain under treatment in special hospitals. The tuberculous patient must be taught to keep still, it may be for many months. This is possible only in special hospitals where the patients must be made to remain as long as the specialists there consider it necessary.

To obtain a standardization of treatment in all hospitals, a course of instruction has been instituted to the great benefit of the patients, and will be given to all the medical officers engaged in the great fight against tuberculosis.

